# Baseline neutrophil-lymphocyte ratio (NLR) predicts toxicity and survival in HR+/HER-2 breast cancer patients treated with CDK4/6 inhibitors

**DOI:** 10.1016/j.breast.2025.104568

**Published:** 2025-08-27

**Authors:** Omar Badran, Ali Darawshe, Sireen Sharif, Samih Yosef, Gil Bar-Sela

**Affiliations:** a Department of Oncology, Emek Medical Center, Afula, postal code 1812201, Israel; b Technion Integrated Cancer Center, Faculty of Medicine, Technion, Haifa, postal code 3125401, Israel; c Research Authority, Emek Medical Center, Afula, postal code 1812201, Israel

**Keywords:** Neutrophil-to-lymphocyte ratio (NLR), CDK4/6 inhibitors, Metastatic breast cancer, Neutropenia, Progression-free survival, Hematologic toxicity

## Abstract

The neutrophil-to-lymphocyte ratio (NLR) is a marker of systemic inflammation that has been associated with prognosis in various malignancies. Its role in predicting toxicity and survival in patients with hormone receptor-positive, human epidermal growth factor receptor 2-negative (HR+/HER2−) metastatic breast cancer treated with cyclin-dependent kinase 4/6 (CDK4/6) inhibitors remains unclear.

**Methods:**

This retrospective cohort study included 2218 patients with HR+/HER2− metastatic breast cancer treated with palbociclib or ribociclib between 2017 and 2024, using data from Israel's largest health maintenance organization. We compared baseline NLR values between patients who developed grade 4 neutropenia (absolute neutrophil count <0.5 × 10^9^/L) and those with higher counts during the first three months of treatment. Additional comparisons were conducted using different neutropenia thresholds. We also assessed the association between baseline NLR (cut-off 2.5), progression-free survival (PFS), and treatment-related adverse events.

**Results:**

Patients with grade 4 neutropenia had significantly higher baseline NLR values compared to those with higher neutrophil counts. The effect size was large in all comparisons. Patients with an NLR of ≥2.5 had a shorter median progression-free survival (PFS) than those with an NLR of <2.5. Hepatotoxicity was more frequently observed in patients with NLR <2.5, while the incidence of dermatologic adverse events was similar across groups.

**Conclusions:**

Elevated baseline NLR is associated with an increased risk of severe neutropenia and shorter progression-free survival in patients treated with CDK4/6 inhibitors. These findings highlight a potential link between systemic inflammation and treatment outcomes, suggesting that NLR may be a valuable predictive biomarker in this setting.

## Highlights

• High baseline NLR is associated with poorer PFS and OS in HR+/HER2− mBC on CDK4/6 inhibitors.
• NLR ≥2.5 is associated with a higher risk of grade 4 neutropenia during treatment.
• Study of over 2,000 patients, one of the largest real-world cohorts assessing NLR in mBC.
• High baseline NLR linked to poorer PFS and OS in HR+/HER2− mBC on CDK4/6 inhibitors.

## 1 Introduction

Breast cancer remains the most diagnosed cancer and the leading cause of cancer-related death among women worldwide [1,2]. Among subtypes, hormone receptor-positive, HER2-negative disease represents the most prevalent group, with a substantial portion progressing to advanced or metastatic stages [3,4]. The introduction of cyclin-dependent kinase 4 and 6 (CDK4/6) inhibitors, specifically, abemaciclib, palbociclib, and ribociclib, has significantly improved treatment outcomes for these patients [[5], [6], [7]]. By inhibiting CDK4/6 activity, these agents induce G1 phase cell cycle arrest in tumor cells [8], leading to prolonged progression-free survival (PFS) and overall survival (OS) [9,10]. Their use in the adjuvant setting has also shown benefit in disease-free and distant recurrence-free survival [11,12].

Despite their clinical efficacy, CDK4/6 inhibitors frequently cause adverse events, most notably neutropenia, the leading dose-limiting toxicity [13]. Unlike chemotherapy-induced neutropenia, which stems from bone marrow suppression, CDK4/6 inhibitor-induced neutropenia is typically nonfebrile, transient, and reversible [14]. Nevertheless, it may require dose reductions or treatment delays, potentially compromising treatment efficacy [[15], [16], [17]].

The neutrophil-to-lymphocyte ratio (NLR) has emerged as a potential biomarker reflecting the balance between systemic inflammation and immune competence [18]. Elevated NLR levels have been associated with a pro-tumor inflammatory microenvironment, driven by neutrophil-mediated tumor promotion and lymphocyte suppression [19,20]. The NLR is an accessible and cost-effective biomarker derived from routine blood counts, and it has shown prognostic and predictive value across a wide range of malignancies, including lung, colorectal, and gastric cancers [21,22]. In breast cancer and other malignancies, high baseline NLR correlates with aggressive tumor behavior, increased metastatic potential, and poorer survival outcomes [[23], [24], [25], [26], [27], [28]], especially in luminal A and HER2-negative subtypes [29]. Conversely, low NLR has been associated with higher pathologic complete response rates following neoadjuvant therapy [30,31]. Post-treatment NLR may also predict recurrence more strongly than baseline levels, particularly in triple-negative breast cancer [32,33]. However, not all studies confirm these associations [30,34].

Beyond prognosis, elevated NLR has been linked to increased risk of febrile neutropenia in chemotherapy-treated patients [[35], [36], [37]], but its role in predicting toxicities associated with CDK4/6 inhibitors remains unclear. Although these agents are known to cause both hematologic and non-hematologic toxicities, such as hepatotoxicity, QT prolongation, and dermatologic events [[38], [39], [40], [41]], data connecting NLR to these outcomes are scarce.

This study aims to assess whether baseline NLR is predictive of CDK4/6 inhibitor-related toxicities and survival outcomes in HR+/HER2− metastatic breast cancer. Identifying patients at increased risk for treatment-limiting toxicities or poor outcomes could support earlier intervention strategies, such as dose adjustments or closer monitoring, and ultimately optimize therapeutic benefit while minimizing harm. Clarifying these relationships could help guide risk stratification and personalize treatment decisions.

## 2 Materials and methods

### 2.1 Study design

This study was a retrospective cohort analysis conducted using data from Clalit Health Services in Northern Israel, the largest healthcare provider in Israel, covering approximately 50 % of the national population [42]. In Northern Israel specifically, Clalit insures around 70 % of individuals enrolled in health maintenance organizations (HMOs) [43], making this dataset highly representative for real-world oncology research.

The study focused on patients diagnosed with hormone receptor-positive (HR+), HER2-negative metastatic breast cancer who received CDK4/6 inhibitors. The availability of comprehensive electronic medical records (EMRs) enabled the collection of robust longitudinal data, including baseline laboratory values, treatment regimens, adverse events, and survival outcomes. This allowed for detailed evaluation of the relationship between baseline neutrophil-to-lymphocyte ratio (NLR), treatment-related toxicities, and progression-free survival in a real-world setting.

### 2.2 Population

Eligible patients were adults (aged 18–99) diagnosed with HR+/HER2− breast cancer who received one of the three approved CDK4/6 inhibitors—abemaciclib, palbociclib, or ribociclib—between January 2017 and December 2024. While all treatment settings were initially included, most patients received CDK4/6 inhibitors for the treatment of metastatic disease. Importantly, abemaciclib is the only agent approved in Israel for adjuvant use, and this indication was added to the national health basket only in January 2024. Because staging data (e.g., metastatic vs. early-stage) were not explicitly available in the dataset, and to ensure that the analysis focused exclusively on patients with metastatic disease, we excluded all patients who received abemaciclib. This decision was made to avoid potential misclassification bias and to preserve cohort homogeneity. In any case, only a small proportion of patients in the database were treated with abemaciclib. The remaining cohort is composed almost entirely of patients treated with palbociclib and ribociclib, and thus reliably represents the population with metastatic HR+/HER2-breast cancer—inclusion criteria required available baseline neutrophil, and lymphocyte counts before therapy initiation.

### 2.3 Data collection

Patient data were extracted from Clalit Health Services' electronic health records (EHR) system. Inclusion criteria encompassed adult patients diagnosed with HR+/HER2− metastatic breast cancer who initiated treatment with either palbociclib or ribociclib between 2017 and 2024; only patients with available baseline laboratory data before CDK4/6 inhibitor initiation were included. Patients receiving abemaciclib were excluded to ensure a uniform cohort of patients with metastatic disease, as abemaciclib is the only CDK4/6 inhibitor approved for adjuvant treatment in the Israeli national health basket.

For each patient, baseline laboratory values, specifically absolute neutrophil count (ANC), lymphocyte count, and the neutrophil-to-lymphocyte ratio (NLR), were collected before CDK4/6 inhibitor therapy. NLR was analyzed as a continuous variable and dichotomized into two groups using a cutoff of 2.5 (<2.5 vs. ≥2.5), which served as a basis for subgroup analysis of toxicity and survival outcomes.

Patients were further stratified based on the severity of hematologic toxicity into two main groups: the severe neutropenia group (ANC <0.5 × 10^9/L) and the Non-Severe neutropenia group (ANC ≥0.5 × 10^9/L). In a complementary analysis, patients were also categorized using a three-tier classification to capture more granular differences: ANC <0.5 × 10^9/L, 0.5–<1.0 × 10^9/L, and ≥1.0 × 10^9/L, enabling a more nuanced assessment of neutropenia severity and its clinical implications.

These two strata formed the core comparative framework for evaluating differences in NLR distribution, treatment characteristics, and clinical outcomes. Among 2218 eligible patients, 70 (3.2 %) developed severe neutropenia (ANC <0.5 × 10^9/L), while 1988 (89.6 %) had ANC values ≥ 0.5 × 10^9/L within the first 3 months of therapy. Of those with ANC ≥0.5, 407 patients had values between 0.5 and <1.0 × 10^9/L, and 1581 had an ANC ≥1.0 × 10^9/L. Additional clinical variables collected included age, gender, Charlson Comorbidity Index score, and type of CDK4/6 inhibitor used. Toxicity endpoints included hepatotoxicity (defined as an elevation in liver enzymes), dermatologic toxicity (characterized by rash and pruritus), and progression-free survival (PFS), which was calculated from the date of treatment initiation to the date of disease progression or death. Due to the lack of available grading information for adverse events, toxicities were analyzed as binary outcomes—categorized simply as present or absent—rather than by severity grade.

Predictive performance of NLR for grade 4 neutropenia was assessed using ROC analysis, and Cliff's delta was used to estimate the effect size of NLR differences between neutropenia groups. All laboratory and clinical data were validated through longitudinal electronic health record (EHR) tracking, and descriptive statistics (median, interquartile range, and range) were calculated for key variables. This comprehensive data structure enabled the investigation of NLR as a predictive biomarker for hematologic toxicity and treatment outcomes in a real-world setting.

### 2.4 Statistical analyses

Descriptive statistics summarized patient demographics, baseline clinical characteristics, laboratory values, and treatment variables. Based on distribution, continuous variables were presented as mean ± standard deviation (SD) or median with interquartile range (IQR). Categorical variables were expressed as counts and percentages.

Group comparisons for categorical variables were conducted using the appropriate chi-square or Fisher's exact test. For non-normally distributed continuous variables, the Mann–Whitney *U* test or the Kruskal–Wallis test was used for more than two groups. Normality was assessed graphically using histograms and Q-plots.

To examine the relationship between NLR and the severity of neutropenia, three pairwise comparisons were performed: patients with an ANC of <0.5 × 10^9/L (grade 4 neutropenia) were compared against those with an ANC between 0.5 × 10^9/L and <1.0 × 10^9/L, those with an ANC of≥1.0 × 10^9/L, and the combined group with an ANC of≥0.5 × 10^9/L. For each comparison, Cliff's delta was used to quantify the effect size of NLR and estimate the likelihood that a patient in the severe neutropenia group would have a higher NLR than those in the comparison group.

Progression-free survival (PFS) was analyzed using the Kaplan–Meier method, and survival curves were compared between patients with a baseline NLR of less than 2.5 and those with a NLR of 2.5 or greater using the log-rank test. A Cox proportional hazards regression model was used to estimate the hazard ratio (HR) and 95 % confidence interval (CI) for progression, adjusting for relevant baseline covariates.

Although NLR was used as a dichotomous variable for subgroup analyses, its predictive ability for grade 4 neutropenia was also evaluated as a continuous variable using a receiver operating characteristic (ROC) curve. The area under the curve (AUC), sensitivity, and specificity were calculated at the optimal threshold determined by the Youden index.

All statistical tests were two-sided; a p-value of less than 0.05 was considered statistically significant. Analyses were performed using R software version 4.1.3 (R Foundation for Statistical Computing, Vienna, Austria).

## 3 Results

Among the 2218 patients included in the analysis, 70 patients (3.2 %) developed severe neutropenia, defined as an absolute neutrophil count (ANC) of less than 0.5 × 10^9/L, during the first three months of CDK4/6 inhibitor therapy. A comparative analysis between this group and patients with an ANC ≥0.5 × 10^9/L (n = 2148) revealed several significant clinical and laboratory differences.

### 3.1 Association between baseline NLR and neutropenia

Patients who developed grade 4 neutropenia had significantly higher baseline neutrophil-to-lymphocyte ratios (NLRs) than those without severe neutropenia (Table 1). Specifically, 61.4 % of patients with an ANC of less than 0.5 had an NLR of 2.5 or greater, whereas only 29.1 % of patients with an ANC of 0.5 or greater had an elevated NLR (p < 0.001). The mean NLR in the severe neutropenia group was 4.73 compared to 2.81 in the non-severe group, with median values of 3.52 vs. 2.25, respectively. Effect size analysis using Cliff's delta yielded a value of 0.497, indicating a significant and clinically meaningful difference in NLR distributions between the groups.

**Table 1:** Patient characteristics by ANC group.

Variable	ANC <0.5 (N = 70)	ANC ≥0.5 (N = 1988)	Total (N = 2058)	P value
NLR (mean ± SD)	4.73 ± 5.88	2.80 ± 2.32	2.86 ± 2.54	<0.001
NLR (median, IQR)	3.52 (2.22–5.15)	2.24 (1.61–3.22)	2.26 (1.61–3.27)	
NLR ≥2.5 (n, %)	43 (61.4 %)	579 (29.1 %)	622 (30.2 %)	<0.001
Age (mean ± SD)	66.56 ± 14.28	64.92 ± 13.86	64.98 ± 13.88	0.330
Age (median, IQR)	68.0 (61–74.75)	66.0 (55–75.0)	66.0 (55–75.0)	
Female (n, %)	68 (97.1 %)	1956 (98.4 %)	2024 (98.3 %)	0.421
Charlson score (mean ± SD)	8.39 ± 3.25	7.13 ± 3.57	7.17 ± 3.57	0.004
Charlson score (median)	8.0	7.0	7.0	
Palbociclib (n, %)	63 (90.0 %)	1632 (82.1 %)	1695 (82.4 %)	0.088
Ribociclib (n, %)	7 (10.0 %)	356 (17.9 %)	363 (17.6 %)	

Age was also significantly higher in patients with grade 4 neutropenia, with a mean of 64.2 years, compared to 61.6 years among patients with an absolute neutrophil count (ANC) ≥ 0.5 (p = 0.031). The Charlson Comorbidity Index, a marker of overall health status, was slightly higher in the severe neutropenia group (mean 8.0 vs. 7.0, p < 0.001), though both values reflect a generally high comorbidity burden. There was no significant difference in sex distribution between the groups, as females comprised 98.6 % of the severe neutropenia group and 98.2 % of the comparison group (p = 0.761).

Regarding treatment, there was no statistically significant difference in the type of CDK4/6 inhibitor used. Ribociclib and palbociclib were evenly distributed among both groups, with 50 % of patients receiving ribociclib (p = 0.918).

Table 1. Baseline characteristics of patients stratified by the severity of neutropenia, comparing those with severe neutropenia (ANC <0.5 × 10^9^/L) to those without (ANC ≥0.5 × 10^9^/L). Variables include age, gender, Charlson comorbidity index, baseline NLR (mean, median, and categorized as <2.5 or ≥2.5), and the type of CDK4/6 inhibitor used.

Further stratification of patients into three groups based on neutrophil counts—ANC <0.5 × 10^9/L (n = 70), ANC 0.5–1.0 × 10^9/L (n = 407), and ANC ≥1.0 × 10^9/L (n = 1581)—demonstrated a clear, statistically significant trend linking lower ANC values with higher baseline NLR. The mean NLR values across the three groups were 4.73 (SD 5.88) for the <0.5 group, 2.34 (SD 1.56) for the 0.5–1.0 group, and 2.10 (SD 1.40) for the ≥1.0 group (p < 0.001). This gradient suggests that the degree of neutropenia correlates with systemic inflammation at baseline, as reflected by elevated NLR. The median NLR values showed a parallel pattern, with medians of 3.52, 1.89, and 1.80 across the three groups.

### 3.2 Impact of NLR on survival outcomes

Based on a predefined threshold of 2.5, the cohort was divided into two subgroups: a low-NLR group (NLR <2.5, n = 1541; 69.5 %) and a high-NLR group (NLR ≥2.5, n = 677; 30.5 %). These stratifications enabled a comparative analysis of survival outcomes between patients with low versus elevated systemic inflammatory status at treatment initiation.

**Fig. 1:**
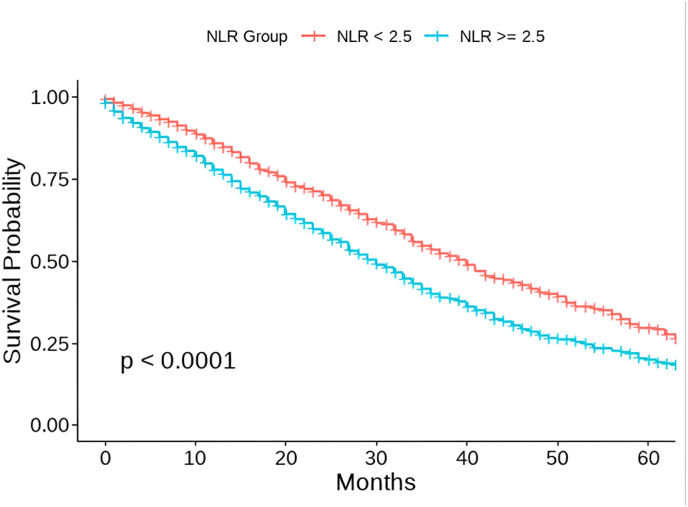
Kaplan–Meier curves for progression-free survival (PFS) stratified by baseline neutrophil-to-lymphocyte ratio (NLR).

Kaplan–Meier survival analysis revealed that patients in the high-NLR group experienced significantly shorter progression-free survival (PFS) compared to those in the low-NLR group (Fig. 1). Median PFS was 24.0 months (IQR: 12–40) for the low-NLR group and 20.0 months (IQR: 10–34) for the high-NLR group (p < 0.001). The hazard ratio (HR) for disease progression or death in patients with a neutrophil-to-lymphocyte ratio (NLR)≥ 2.5 was 1.27 (95 % CI: 1.14–1.42), indicating a 27 % increased risk compared to patients with lower NLR values. The estimated five-year progression-free survival (PFS) further highlighted this gap, with 26.0 % of patients in the low-NLR group remaining progression-free at 5 years, compared to only 16.9 % in the high-NLR group.

Fig. 1. Kaplan–Meier curves of progression-free survival (PFS) in patients with HR+/HER2− metastatic breast cancer treated with CDK4/6 inhibitors, stratified by baseline neutrophil-to-lymphocyte ratio (NLR <2.5 vs. NLR ≥2.5). The red line represents patients with an NLR of less than 2.5, and the blue line represents patients with an NLR of 2.5 or greater.

Similarly, a statistically significant difference was observed in overall survival (OS). The three-year overall survival (OS) rate was 29.9 % in the low NLR group, compared to 22.1 % in the high NLR group. The HR for death in the high-NLR group was 1.27 (95 % CI: 1.13–1.43; p < 0.001), supporting the negative prognostic value of elevated baseline NLR.

### 3.3 Association of NLR with treatment-related toxicities

Treatment-related adverse events were compared between the low-NLR (<2.5) and high-NLR (≥2.5) groups to assess potential associations between systemic inflammation and treatment toxicity profiles (Table 2). Hepatotoxicity, defined as elevated liver enzymes, occurred more frequently in the low-NLR group compared to the high-NLR group. Specifically, 11.8 % (182/1541) of patients with an NLR of less than 2.5 developed hepatotoxicity, compared to 7.4 % (50/677) of those with an NLR of 2.5 or greater, a statistically significant difference (p = 0.002).

**Table 2:** Adverse events by baseline neutrophil-to-lymphocyte ratio (NLR) group.

Adverse Event	NLR <2.5	NLR ≥2.5	p-value
Elevated Liver Enzymes	218 (12.4 %)	63 (8.3 %)	0.003
Rash	164 (9.3 %)	54 (7.1 %)	0.069
Pruritus	174 (9.9 %)	74 (9.7 %)	0.902

Table 2. Incidence of selected treatment-related adverse events among patients treated with endocrine therapy and CDK4/6 inhibitors, stratified by baseline neutrophil-to-lymphocyte ratio (NLR <2.5 vs. NLR ≥2.5). The table includes the frequencies and percentages of elevated liver enzymes, rash, and pruritus in each group.

Dermatologic toxicities, including rash and pruritus, did not significantly differ between the two NLR groups. Rash was reported in 9.0 % of patients with an NLR of less than 2.5 and in 6.8 % of those with an NLR of 2.5 or greater (p = 0.081). Pruritus occurred in 9.9 % of both groups (p = 0.981), indicating no meaningful difference in skin-related adverse effects based on baseline NLR. QT interval prolongation, a known adverse effect associated particularly with ribociclib, was too infrequent in the cohort to allow for a robust statistical comparison between NLR groups.

## 4 Discussion

The neutrophil-to-lymphocyte ratio (NLR) has been increasingly recognized as a significant biomarker in oncology, reflecting the balance between systemic inflammation and immune response [[44], [45], [46], [47]]. In the context of hormone receptor-positive (HR+)/HER-2 negative breast cancer treated with CDK4/6 inhibitors, our study adds to the growing body of evidence supporting the predictive and prognostic value of NLR. One of the primary findings of our study was the consistent association between elevated baseline NLR and the development of severe neutropenia during CDK4/6 inhibitor therapy. This pattern held across multiple stratifications of neutrophil count, revealing a clear trend: patients with lower absolute neutrophil counts following treatment were more likely to have presented with higher baseline NLR. This reinforces the hypothesis that a pro-inflammatory state indicated by elevated NLR may reflect a more fragile hematopoietic reserve or an altered marrow environment that predisposes patients to hematologic toxicity under cytostatic stress.

What is particularly noteworthy is that this association remained robust after accounting for key clinical variables such as age, comorbidity burden, and type of CDK4/6 inhibitor used. This suggests that the relationship between NLR and severe neutropenia is not merely confounded by baseline patient frailty or treatment choice but may instead reflect an intrinsic biological vulnerability. It raises the possibility that NLR can serve as a simple, pre-treatment tool to identify patients at higher risk for dose-limiting toxicities, allowing for more proactive monitoring or early dose adjustments. The biological rationale for this association lies in the pro-inflammatory state reflected by elevated NLR, which may impair hematopoiesis or disrupt the bone marrow microenvironment, ultimately making patients more vulnerable to cdk 4/6 -induced myelosuppression [[48], [49], [50]].

Building on these findings, our study demonstrated that elevated NLR was associated with worse progression-free and overall survival. This aligns with prior literature describing NLR as a prognostic marker in breast cancer and other malignancies [[50], [51], [52], [53], [54]]. Elevated NLR is believed to reflect a microenvironment dominated by neutrophil-mediated tumor promotion and suppressed lymphocyte-mediated tumor surveillance. In the context of CDK4/6 inhibition, where immune modulation plays a secondary yet relevant role, these immunologic imbalances may contribute to shorter disease control and reduced treatment efficacy.

The consistent association between high NLR (≥2.5) and inferior clinical outcomes, as reflected in median PFS and 3-year survival probabilities, aligns with prior literature linking systemic inflammation to tumor progression, immune suppression, and resistance to therapy [[55], [56], [57], [58], [59], [60]]. However, the 3-year overall survival observed in our real-world cohort was notably lower compared to the outcomes reported in randomized prospective clinical trials such as MONALEESA-2, MONALEESA-3, and PALOMA-3 [61,62]. Several factors may account for this survival gap. Unlike the controlled environment of clinical trials, real-world populations tend to be more heterogeneous and medically complex, often including older patients, individuals with multiple comorbidities, and those with reduced performance status. Our cohort included both patients who initiated CDK4/6 inhibitors as first-line therapy for de novo metastatic disease and those who experienced recurrence after prior adjuvant endocrine therapy. However, before the Israeli national health basket fully reimbursed CDK4/6 inhibitors in 2018, many patients were initially managed with endocrine therapy alone and only later escalated to CDK4/6 inhibitors. This treatment delay may have introduced lead-time bias, resulting in shorter observed survival from the point of CDK4/6 initiation compared to clinical trials, where therapy typically began at the time of metastatic diagnosis.

The hepatotoxicity is a well-recognized adverse event associated with CDK4/6 inhibitors, with varying incidence rates reported across clinical trials. In the MONALEESA-2 trial, abnormal liver function tests, including elevated ALT, AST, and bilirubin, were reported in 20.1 % of patients (all grades), with Grade 3 and 4 events occurring in 8.4 % and 1.8 %, respectively [6]. The MONALEESA-3 trial reported abnormal liver function test (LFTs) in 25.4 % of patients, with Grade 3 and 4 elevations in 8.5 % and 1.3 %, respectively [61]. Similarly, in PALOMA-3, AST elevations occurred in 11.6 % of patients (all grades), including 3.2 % with Grade 3 toxicity, with no Grade 4 events [62].

Interestingly, our analysis revealed an inverse association between NLR and hepatotoxicity. Patients with lower NLR values exhibited significantly higher rates of liver enzyme elevation. While the underlying mechanism remains speculative, it is plausible that differential hepatic immune responses or metabolic profiles may underlie this observation.

In contrast, dermatologic toxicities such as rash and pruritus did not correlate with NLR levels. This supports the notion that these adverse effects are likely unrelated to systemic inflammatory status and more attributable to direct pharmacologic or skin-specific pathways. These findings are consistent with prior reports that describe a spectrum of cutaneous adverse reactions, many of which appear to be drug-specific. However, the underlying mechanisms remain unclear, and whether these toxicities are linked to immune or inflammatory status is not yet established [63]. These findings suggest that NLR plays a dual role as both a prognostic and toxicity-related biomarker. While it may not replace molecular or genomic tools, its simplicity, accessibility, and reproducibility make it attractive for integration into routine clinical workflows. Future prospective studies are needed to explore whether dynamic monitoring of NLR during treatment can further refine prognostication or guide real-time therapeutic decisions.

The strengths of this study include its large, real-world cohort and the comprehensive evaluation of both clinical outcomes and treatment-related toxicities. Including multiple layers of comparison, including NLR and ANC levels stratification, adds depth to the analysis and helps distinguish between prognostic and predictive implications. However, the retrospective design introduces potential limitations, including unmeasured confounding and lack of external validation.

## 5 Conclusions

In this large, real-world cohort of patients with HR+/HER-2 metastatic breast cancer treated with CDK4/6 inhibitors, baseline neutrophil-to-lymphocyte ratio (NLR) emerged as a clinically relevant biomarker for both hematologic toxicity and survival outcomes. Elevated NLR was significantly associated with the development of grade 4 neutropenia, even after adjusting for age, comorbidities, and treatment type. These findings suggest that a pro-inflammatory host environment may be associated with increased bone marrow vulnerability during treatment. Additionally, patients with an NLR of ≥2.5 tended to experience shorter progression-free and overall survival, suggesting a potential prognostic role for NLR that warrants further investigation.

## CRediT authorship contribution statement

**Omar Badran:** Writing – review & editing, Writing – original draft, Validation, Investigation, Formal analysis, Data curation, Conceptualization. **Ali Darawshe:** Writing – original draft, Conceptualization. **Sireen Sharif:** Validation, Software. **Samih Yosef:** Writing – original draft, Conceptualization. **Gil Bar-Sela:** Writing – review & editing, Validation, Supervision, Funding acquisition.

## Informed consent statement

Patient consent was waived due to the study's retrospective nature and the use of anonymized data, as approved by the Institutional Review Board of Emek Medical Center (protocol code EMC-0095-24).

## Institutional review board statement

The study was conducted by the Declaration of Helsinki and approved by the Institutional Review Board of Emek Medical Center (protocol code EMC-0095-24, approval date: March 7, 2024).

## Funding

This research received no external funding. The authors funded the APC.

## Declaration of competing interests

The authors declare that they have no known competing financial interests or personal relationships that could have appeared to influence the work reported in this paper.
